# Comparison of Water Sorption and Solubility of Conventional and Bulk-Fill Composites at Different Depths Over Two Time Intervals

**DOI:** 10.7759/cureus.89150

**Published:** 2025-07-31

**Authors:** Nafise Elmamooz, Ali Eskandarizadeh, Jahangir Haghani, Zahra Doosty, Faranak Rahimi, Maryam Shakerifar

**Affiliations:** 1 Department of Restorative Dentistry, School of Dentistry, Kerman University of Medical Sciences, Kerman, IRN; 2 Department of Oral and Maxillofacial Radiology, School of Dentistry, Kerman University of Medical Sciences, Kerman, IRN; 3 Department of Restorative Dentistry, Private Practice, Tehran, IRN; 4 Department of Restorative Dentistry, School of Dentistry, Islamic Azad University, Tehran, IRN

**Keywords:** bulk-fill, composite resin, solubility, time interval, water sorption

## Abstract

Background and aim: Bulk-fill composites are a class of restorative materials introduced to speed up the restoration process in the posterior area. This study aimed to compare the water sorption and solubility of a bulk-fill resin composite with its conventional counterpart at various depths after one week and one month of water storage.

Methods: Five cylindrical specimens (15 × 8 mm) of each composite resin, Tetric N Ceram Bulk fill and Tetric N Ceram (Schaan, Liechtenstein: Ivoclar Vivadent), were prepared using a metal split mold. The conventional composite was placed incrementally in 2 mm layers, while the bulk-fill composite was applied in a single layer. Specimens were cured and polished, then cut into disc-shaped samples (1 mm thickness and 15 mm diameter) (n=40, five disks per depth for each composite). Water sorption and solubility were assessed after one week and one month of immersion in distilled water at 37±1 °C. Statistical analysis was performed using repeated measures analysis of variance and t-test (p≤0.05).

Results: The bulk-fill composite exhibited significantly higher water sorption and solubility than the conventional composite at all depths measured. Water sorption and solubility increased with depth and were significantly greater after one month compared to one week.

Conclusion: The bulk-fill composite demonstrated higher water sorption and solubility compared to the conventional composite, with significant variations observed at different depths over time. A conventional composite may work better in terms of water sorption and solubility, and must be considered in extensive posterior restorations.

## Introduction

Significant advances in dental composite resin materials, coupled with increasing patient demand for tooth-colored restorations, have led to the growing popularity of these materials for posterior restorations. However, the limited depth of light penetration in conventional composite resins poses a challenge, particularly regarding polymerization shrinkage in larger cavities [1,2].

The incremental method is a standard technique for placing composite resins in cavities deeper than 2 mm, applying triangular or horizontal layers with a maximum thickness of 2 mm per layer [3]. The rationale behind this technique is to limit the thickness of the composite resin, thereby enhancing light penetration and reducing polymerization shrinkage [4]. However, employing this technique in deep cavities can be time-consuming, and there is an increased risk of bubble incorporation and contamination between layers. Therefore, it is essential to explore faster and more efficient restoration methods that maintain appropriate physical properties [5,6].

In this context, bulk-fill composite resins have emerged, with manufacturers claiming that they can be applied in layers of 4-5 mm thickness [7-9]. These materials reduce the number of layers required to fill a cavity compared to the conventional layering technique, simplifying the restoration process and decreasing clinical time. Bulk-fill composites exhibit improved polymerization, more controlled shrinkage stress, and reduced cuspal flexion compared to conventional composites [3].

The oral cavity presents a challenging environment for restorative materials, making resistance to degradation a critical factor in their clinical durability. Water sorption in composite resins occurs through a diffusion-controlled mechanism, leading to chemical degradation due to the release of unreacted monomers and debonding at the filler-matrix interface [10]. This process diminishes the mechanical and physical properties of composite resins, including strength, hardness, wear resistance, color, and dimensional stability. Additionally, solubility can influence the biocompatibility of materials, leading to volume reduction and weakening [6,11,12]. The water sorption and solubility properties of composite resins are influenced by their formulation, including the volume, size, shape, and spacing of the filler particles, the type of monomer used, the degree of curing, and the quality of the filler-matrix bond [6,12]. Investigating these parameters is crucial for determining the long-term success of restorative materials [2].

Since bulk-fill composite resins can be applied in thicknesses of 4-5 mm, their properties at greater depths may vary with distance from the surface and the light source. Therefore, this study aimed to investigate and compare the water sorption and solubility properties of Tetric N Ceram and Tetric N Ceram conventional composite resins after one week and one month of storage in water at different depths. The null hypotheses were as follows: water sorption and solubility of the two composites would not differ at different depths over the time intervals, and water sorption and solubility of the bulk-fill composite would not differ from those of their conventional counterparts.

## Materials and methods

In this experimental study (ethics code: IR.KMU.REC.1394.08), two commercial resin composites were used as follows: a conventional composite, Tetric N Ceram (Schaan, Liechtenstein: Ivoclar Vivadent) (shade A2), and a bulk-fill composite, Tetric N Ceram Bulk fill (Schaan, Liechtenstein: Ivoclar Vivadent) (shade IVA) (Table 1). Five cylindrical specimens (15 × 8 mm) were fabricated using a scaled two-piece stainless-steel mold from each of the resin composites. The composites were applied and cured according to the manufacturer's instructions. Tetric N Ceram was placed incrementally in layers of 2 mm, with each layer cured for 20 s. Tetric N Ceram Bulk fill was applied in two layers of 4 mm and each layer was cured for 20 s. Curing was performed using a light-emitting diode Demi Ultra (Brea, CA: Kerr; 1200 mW/cm²) from top surface of the mold at three overlapping sections through a glass slide. The tip of curing device was kept in contact with glass slide during curing. The output of the curing device was periodically verified with a radiometer (Brea, CA: Kerr).

**Table 1:** Materials specifications. Bis-GMA: bisphenol A-glycidyl methacrylate; Bis-EMA: ethoxylated bisphenol A dimethacrylate; UDMA: urethane dimethacrylate

Composite	Type	Manufacture	Batch no./shade	Composition
Tetric N Ceram	Conventional	Schaan, Liechtenstein: Ivoclar Vivadent	U20277, A2	Resin: Bis-GMA, UDMA filler: Ba glass, Yb3f, mixed oxide (80-81 wt%)
Tetric N Ceram Bulk fill	High viscosity bulk-fill	Schaan, Liechtenstein: Ivoclar Vivadent	U03089, IVA	Resin: Bis-GMA, UDMA filler: BA-AL-Si glass prepolymerized filler (monomer, glass filler, Ybf) mixed oxide (75-77 wt%)

After that, the cylindrical samples were sectioned perpendicularly to the long axis using a handpiece (Marathon, N1; South Korea, Daegu: Saeyang Microtech Co., Ltd.) and a diamond disc (Centennial, CO: Hager & Meisinger GmbH) under continuous water cooling to supply disc-shaped 1 mm thick specimens, in accordance with ISO:4049:2019 [13]. A digital caliper (Kawasaki, Japan: Mitutoyo Corporation) was used to check sample dimensions. Five specimens were obtained per depth of 1, 2, 3, and 4 mm, resulting in a total of 20 specimens for each composite at various depths (total=40) [14]. The specimens were placed in individual vials containing silica gel and stored in a desiccator for 22 h at a temperature of 37±1°C, followed by an additional 2 h at 23±1°C, in accordance with ISO 4049 guidelines. After incubation period, the specimens were weighed daily using a digital balance GR-202 (Tokyo, Japan: A&D Company, Limited) with an accuracy of 0.01 mg until a constant mass (m_1_) was achieved. The thickness and diameter of the discs were measured at three points using a digital caliper (Kawasaki, Japan: Mitutoyo Corporation), and the volume of each specimen was calculated. The specimens were then individually placed in a glass container, immersed in at least 10 mL of distilled water, and incubated at 37±1°C. At seven-day and one-month intervals, specimens were dried with filter paper and gentle airflow, weighed, and returned to the water. The mass acquired at this stage was defined as m_2_. The storage water was changed every 24 h to maintain consistent pH levels. At the end of each storage period, the specimens were placed in a desiccator containing fresh silica gel to achieve a stable weight (m_3_). Solubility and water sorption were calculated using the following formulas in micrograms per cubic millimeter (µg/mm³).

$\text{Water sorption (WSP)} = \frac{m_2 - m_3}{v}$

$\text{Water solubility (WSL)} = \frac{m_1 - m_3}{v}$

Here, m_1_ is the mass before water storage, m_2_ is the mass after water storage for seven days or one month, m_3_ is the mass after the desorption cycle, and v is the specimen’s volume [15].

Statistical analysis

Data were analyzed using SPSS software version 27 (Armonk, NY: IBM Corp.) with a significance level set at p≤0.05. Descriptive indices (mean, standard deviation) were used to describe the water sorption and solubility values. Normality was checked using the Kolmogorov-Smirnov test and revealed normal distribution between the values of the groups. An independent samples t-test was utilized to compare W_SP_ and W_SL_ values of two composites and two time intervals at any depth, and a paired t-test was employed to analyze any composite at different depths. W_SP_ and W_SL_ values at different depths at any time intervals were analyzed by repeated measures analysis of variance.

## Results

Means and standard deviations of water sorption and solubility of two composites at two time intervals are presented in Tables 2, 3. Based on Table 2, water sorption of bulk-fill composite was significantly higher than that of conventional composite at all depths over both time intervals (p≤0.05).

**Table 2:** Comparison of water sorption of conventional and bulk-fill composites during one week and one month. *Independent t-test between two composites. **Independent t-test between two times.

Depth	Composite	Mean 1 week	SD 1 week	Mean 1 month	SD 1 month	p-Value**
1 mm	Bulk fill	32.84	0.67	46.51	1.01	0.0001
	Conventional	24.71	0.82	27.77	1.10	0.0001
	P-value*	0.0001		0.0001		-
2 mm	Bulk fill	33.67	0.87	68.82	0.87	0.0001
	Conventional	25.34	0.91	31.72	1.40	0.0001
	P-value*	0.0001		0.0001		-
3 mm	Bulk fill	33.87	0.36	70.39	1.72	0.0001
	Conventional	27.57	0.78	32.54	1.68	0.001
	P-value*	0.0001		0.0001		-
4 mm	Bulk fill	35.04	0.88	71.19	2.30	0.0001
	Conventional	31.65	1.16	32.84	0.75	0.09
	P-value*	0.001		0.0001		-

**Table 3:** Comparing the solubility of conventional and bulk-fill composites during one week and one month. *Independent t-test between two composites. **Independent t-test between two times.

Depth	Composite	Mean 1 week	SD 1 week	Mean 1 month	SD 1 month	p-Value**
1 mm	Bulk fill	2.55	0.28	15.24	0.45	0.0001
	Conventional	2.15	0.24	4.11	0.25	0.0001
	P-value*	0.04		0.0001		-
2 mm	Bulk fill	2.81	0.51	16.38	0.47	0.0001
	Conventional	2.57	0.27	4.35	0.21	0.0001
	P-value*	0.38		0.0001		-
3 mm	Bulk fill	2.99	0.42	18.25	0.33	0.0001
	Conventional	2.62	0.11	4.63	0.12	0.0001
	P-value*	0.12		0.0001		-
4 mm	Bulk fill	3.19	0.34	19.50	0.41	0.0001
	Conventional	2.66	0.40	4.88	0.13	0.0001
	P-value*	0.05		0.0001		-

Additionally, the water sorption of the two composites at one month was significantly higher than at one week at all depths, except at a depth of 4 mm for the conventional composite, which was not statistically significant (Table 2). Solubility characteristic of bulk-fill composite was significantly greater at all depths over two time intervals compared to conventional composite one, except for depths of 2 and 3 mm in a one-week period, which was not significant. The solubility of the two composites was significantly higher after one month than after one week at all depths (Table 3).

**Figure 1:**
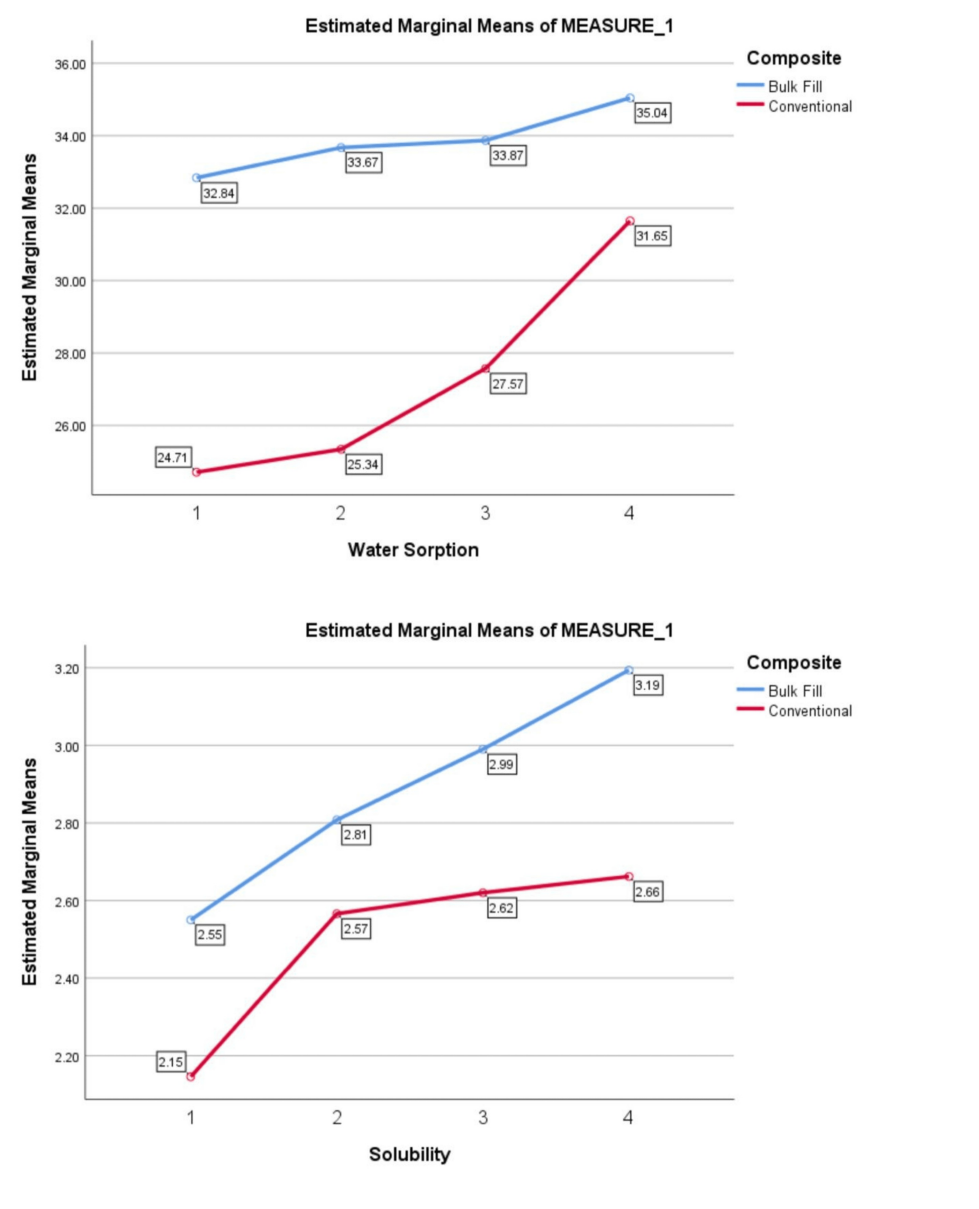
Trends in changes in water sorption and solubility between different depths in one week in two composites.

**Figure 2:**
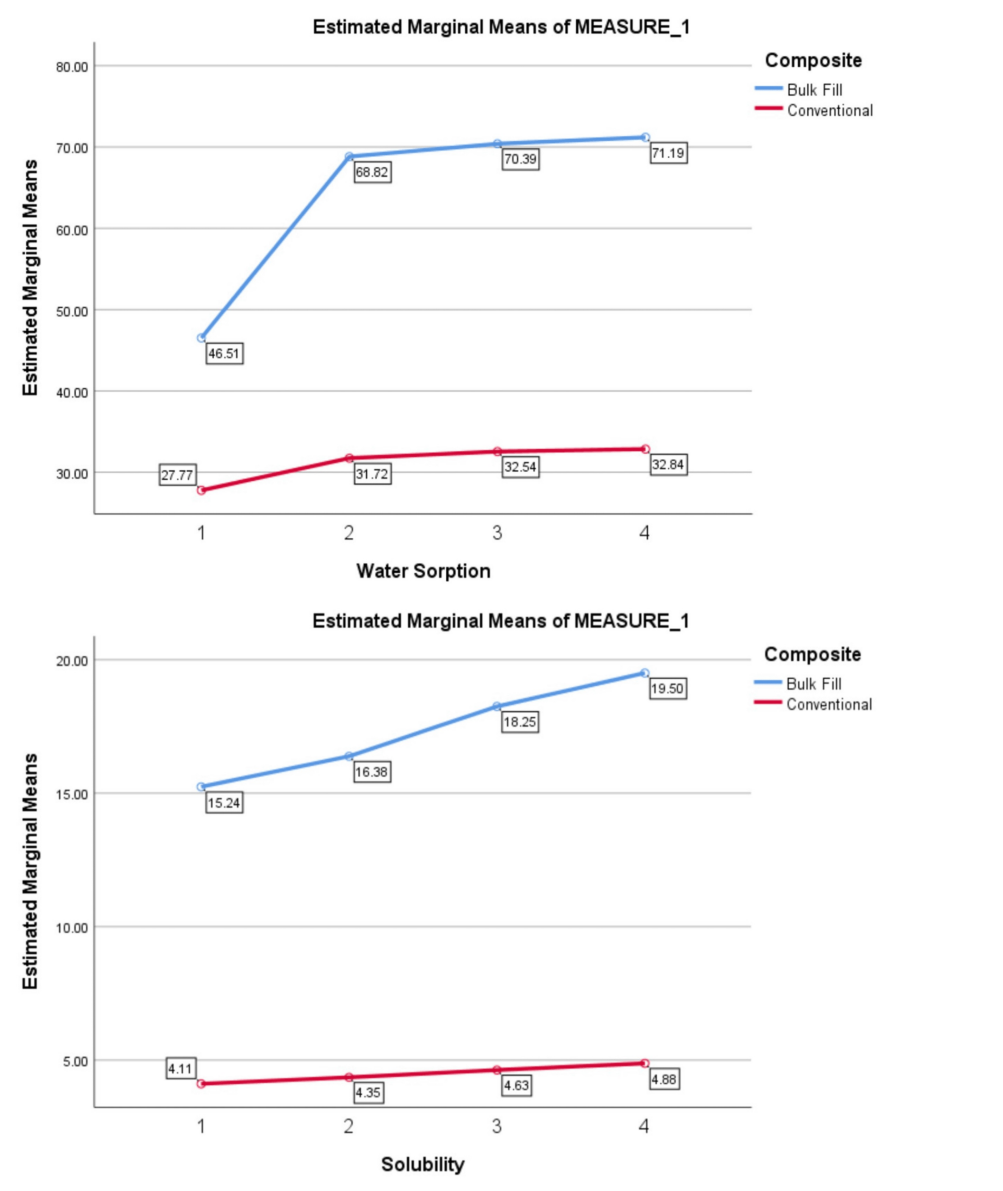
Trends in changes in water sorption and solubility between different depths in one month in two composites.

Trends in water sorption and solubility across different depths over one week and one month are illustrated in Figures 1, 2. According to the repeated measures test, the overall trends in water sorption and solubility significantly increased in both types of composite with depth at both time intervals (p=0.001).

Pairwise comparisons of water sorption and solubility within different groups at different depths are shown in Tables 4, 5.

**Table 4:** Pairwise comparisons of water sorption and solubility of conventional composite between different depths. *Paired t-test.

Variables	Depth	1 Week			1 Month		
		Mean difference	SD	p-Value*	Mean difference	SD	p-Value*
Water sorption	1-2 mm	-0.63	1.11	0.27	-3.95	0.69	0.0001
	1-3 mm	-2.86	1.51	0.01	-4.77	1.00	0.0001
	1-4 mm	-6.94	1.13	0.01	-5.07	0.81	0.0001
	2-3 mm	-2.23	1.31	0.02	-0.82	0.33	0.005
	2-4 mm	-6.31	1.61	0.001	-1.12	0.88	0.05
	3-4 mm	-4.08	1.78	0.007	-0.30	1.13	0.58
Solubility	1-2 mm	-0.42	0.25	0.02	-0.24	0.31	0.16
	1-3 mm	-0.47	0.23	0.01	-0.52	0.25	0.01
	1-4 mm	-0.52	0.31	0.02	-0.77	0.29	0.004
	2-3 mm	-0.05	0.21	0.60	-0.27	0.17	0.02
	2-4 mm	-0.10	0.55	0.72	-0.53	0.28	0.01
	3-4 mm	-0.04	0.45	0.84	-0.25	0.12	0.01

**Table 5:** Pairwise comparisons of water sorption and solubility of bulk fill composite between different depths. *Paired t-test.

Variables	Depth	1 Week			1 Month		
		Mean difference	SD	p-Value*	Mean difference	SD	p-Value*
Water sorption	1-2 mm	-0.63	1.11	0.12	-22.31	0.85	0.0001
	1-3 mm	-2.86	1.51	0.05	-23.88	1.55	0.0001
	1-4 mm	-6.94	1.13	0.02	-24.68	1.41	0.0001
	2-3 mm	-2.23	1.31	0.73	-1.57	1.54	0.09
	2-4 mm	-6.31	1.61	0.08	-2.37	2.03	0.06
	3-4 mm	-4.08	1.78	0.05	-0.80	1.67	0.35
Solubility	1-2 mm	-0.42	0.25	0.37	-1.14	0.77	0.03
	1-3 mm	-0.47	0.23	0.05	-3.01	0.47	0.0001
	1-4 mm	-0.52	0.31	0.02	-4.26	0.61	0.0001
	2-3 mm	-0.05	0.21	0.48	-1.87	0.75	0.005
	2-4 mm	-0.10	0.55	0.03	-3.12	0.36	0.0001
	3-4 mm	-0.04	0.45	0.20	-1.25	0.72	0.02

## Discussion

The integrity of dental composite resins in the oral environment is significantly influenced by water sorption and solubility, which depend on chemical composition and polarity of molecular structure, and the quality of polymerization. Following water sorption, hygroscopic expansion occurs, leading to the release of unreacted monomers [14,16].

In this study, distilled water was used to simulate the aqueous environment of the mouth for assessing water sorption and solubility, consistent with previous studies [11,16]. It has been shown that maximum water sorption typically occurs within the first month of water storage, with structural changes and decreases in physical properties primarily occurring within this period, so this study was designed to investigate these properties at one-week and one-month intervals [17,18].

Bulk-fill composite resins contain polymerization modulators or plasticizers in their resin matrix, which help mitigate polymerization shrinkage stress when the material is placed in bulk. These chemical modifications enhance the quality of the polymer network and improve moisture resistance compared to conventional composite resins [14,19].

The water sorption and solubility values in the Tetric N Ceram conventional composite resin at both time intervals (one week and one month) across all depths fell within the standard range established by ISO 4049 (≤40 µg/mm³ for water sorption and ≤7.5 µg/mm³ for solubility) [13]. However, for the Tetric N Ceram Bulk fill composite resin, these parameters exceeded ISO 4049 standards at the one-month interval across all depths.

The first null hypothesis was rejected. A significant difference in water sorption and solubility of the composite resins studied was observed between the two time intervals, consistent with previous research indicating that these properties are time-dependent phenomena that increase over time [8,20, 21]. The current study demonstrated that as depth increased, water sorption and solubility also increased for both composite resin groups, aligning with prior studies [22-24]. This trend can be attributed to a decrease in the degree of polymerization conversion with increasing depth, as light intensity diminishes with greater thickness. Consequently, reduced light intensity affects the degree of conversion, leading to increased water sorption and solubility [25]. A strong relationship exists between the degree of conversion of polymers and the characteristics of the polymer network, including their hydrophilicity [22,26]. Thus, measuring water sorption and solubility can serve as an indirect method to estimate the polymerization quality of composite resins [8]. The solubility of dental composite resins is influenced by the release of unreacted monomers during curing; a reduction in the degree of conversion in deeper areas leads to an increase in unreacted monomers and, subsequently, higher solubility [11,20].

The second null hypothesis was also rejected. In this study, Tetric N Ceram Bulk fill composite resin exhibited significantly higher water sorption and solubility at all depths compared to the conventional Tetric N Ceram composite resin. Previous studies on the water sorption and solubility of bulk-fill composite resins have yielded conflicting results, possibly due to differences in procedural steps [6,9,11,14]. Some studies reported no significant differences in water sorption between the two types of composite resins [27,28], while others indicated that bulk-fill resins had lower water sorption than conventional composites [2,14]. The composition of the polymer matrix and hydrophobicity, the size and content of filler particles, and the chemistry of the silane agent can influence the water sorption and solubility [7,11,28]. Both conventional Tetric N Ceram and Tetric N Ceram Bulk fill composites have a similar resin matrix composition, comprising bisphenol A-glycidyl methacrylate (Bis-GMA), ethoxylated bisphenol A dimethacrylate (Bis-EMA), and urethane dimethacrylate (UDMA). However, the filler content in bulk-fill composite resin is lower than that in the conventional composite resin (75-77% vs. 80-81%) [29]. Water sorption predominantly occurs in the resin matrix, and previous studies have demonstrated an inverse relationship between filler content and the amount of water sorption; as filler content increases, the relative amount of the polymer matrix decreases, leading to lower water sorption and solubility in the conventional composite [11,14]. Additionally, the presence of prepolymerized fillers in the Tetric N Ceram Bulk fill composite resin may promote water sorption due to the polymerized hydrophilic functional groups, further justifying the increased water sorption observed in this material compared to the conventional composite [14,30].

As an in vitro study, this research may not completely mimic the clinical condition of oral environment in terms of intervening chemical and mechanical factors. Another issue is the limited water storage period of the study. Longer periods of water immersion might reveal more reliable information. In this study, only one brand of composite was tested, so we suggest evaluating and employing multiple brands and formulations of composites in clinical trials.

## Conclusions

Within the limitations of this study, the results indicate that water sorption and solubility values for the bulk-fill composite were significantly higher than those for the conventional composite. Notably, the differences in both types of composites at varying depths were significant after one month compared to one week. Furthermore, increased depth correlated with higher values of water sorption and solubility in both composites.
